# Renal dysfunction is associated with decline of cognitive function in community-dwelling older adults: Korean frailty and aging cohort study

**DOI:** 10.1186/s12877-020-01862-8

**Published:** 2020-11-10

**Authors:** Ji Yoon Kong, Jin Sug Kim, Min Hye Kang, Hyeon Seok Hwang, Chang Won Won, Kyung Hwan Jeong

**Affiliations:** 1 Department of Medicine, Graduate School, Kyung Hee University, Seoul, Republic of Korea; 2 Division of Nephrology, Department of Internal Medicine, College of Medicine, Kyung Hee University, Seoul, Republic of Korea; 3 Department of Family Medicine, College of Medicine, Kyung Hee University, Seoul, Republic of Korea

**Keywords:** Cognitive function, Cohort study, Older adults, Renal function

## Abstract

**Background:**

Cognitive decline is common in older adults. Similarly, the prevalence of renal dysfunction is also increased in the elderly population. We conducted this study to clarify the relationship between renal dysfunction and decline of cognitive function in community-dwelling elderly population.

**Methods:**

A cross-sectional analysis was performed using data from the Korean Frailty and Aging Cohort Study, a nationwide cohort study. Total 2847 (1333 men, 1514 women) eligible participants were enrolled for this study. The estimated glomerular filtration rate (eGFR, mL/min/1.73m^2^) was calculated using the Chronic Kidney Disease Epidemiology Collaboration equation. Global cognitive function was assessed with the Mini-mental State Examination-Korean version. Other domains of cognitive function were tested with the Consortium to Establish a Registry for Alzheimer’s disease and the Frontal Assessment Battery.

**Results:**

The mean age of all participants was 76.0 ± 3.9 years and eGFR (all in mL/min/1.73 m^2^) was 77.5 ± 14.3. And the mean eGFR was 91.7 ± 3.2 in quartile 1, 84.9 ± 1.8 in quartile 2, 76.1 ± 3.7 in quartile 3, and 57.2 ± 10.8 in quartile 4. In baseline characteristics, participants with lower eGFR tend to have lower cognitive function scores than participant with higher eGFR. In linear regression analysis, eGFR was correlated with the word list memory (β = 0.53, *P* = 0.005), word list recall (β = 0.86, *P* < 0.001), and word list recognition (β = 0.43, *P* = 0.030) after adjustment of confounding variables. Moreover, after multivariate adjustment the association with cognitive impairment in quartile 2 was stronger (adjusted OR: 1.535, 95% CI: 1.111–2.120, *P* = 0.009), and the ORs of cognitive impairment were 1.501 (95% CI: 1.084–2.079, *P* = 0.014) in quartile 3 and 1.423 (95% CI: 1.022–1.983, *P* = 0.037) in quartile 4.

**Conclusion:**

In older adults, the immediate, recent memory, and recognition domains were significantly related to renal function. Also, the mild renal dysfunction was independently associated with impairment of global cognitive function. These results suggest that the early stages of renal dysfunction could be an effective target to prevent worsening of cognitive impairment. Therefore, regular monitoring and early detection of mild renal dysfunction in elderly population might be needed.

**Supplementary Information:**

The online version contains supplementary material available at 10.1186/s12877-020-01862-8.

## Background

Renal dysfunction is common in older adults and strongly related to the risk of cardiovascular and cerebrovascular diseases [1, 2]. Further, the prevalence of cognitive impairment and dementia, which can increase the social and economic burden and reduce the quality of life, is higher in the elderly population [3, 4].

To date, many cross-sectional or longitudinal studies have been performed to determine the relationship between renal function and cognitive function. Patients with chronic kidney disease (CKD) and end-stage renal disease (ESRD) on hemodialysis have a high risk of cognitive impairment [5, 6]. Moreover, among patients with estimated glomerular filtration rate (eGFR) less than 60 mL/min/1.73m^2^, those with lower eGFR have a higher risk of cognitive impairment [7]. In recent meta-analysis studies, CKD was determined to be an independent somatic risk factor for the development of cognitive decline [8], and cognitive changes were found to occur early in CKD with respect to the orientation-attention and language domains [9]. Moreover, in prior studies of the elderly populations, CKD was found to increase the incidence of cognitive impairment and vascular dementia [10–15]. And cognitive decline was also found to be more rapid in patients with CKD [16].

Most of the previous studies have focused on patients with moderate to severe renal dysfunction including ESRD, whereas few studies have focused on patients with mild renal dysfunction, which takes more than half percentage of all-stage CKD patients [17]. The global prevalence of all-stage CKD, showed differences by literature, was estimated as 8.5–9.8% and 697.5 million cases were reported worldwide [18–20]. Moreover, the mild renal dysfunction in CKD stage 1 and 2, was accounted for 5.0%. Likewise, in Korea, the estimated prevalence of all-stage CKD was 8.2%, and about 5.7% was included in stage 1 and 2 [21]. Further, it remains unclear whether renal function is associated with function of different cognitive domains in the community-dwelling older adult population.

The Korean Frailty and Aging Cohort Study (KFACS) is a multicenter, longitudinal study that began in 2016. The participants were community-dwelling older adults aged 70–84 years who were recruited from urban and rural regions across Korea. Other details of the KFACS have been previously described [22, 23]. Recently, KFACS data have demonstrated the relationship between cognitive function and calf circumference, sarcopenia, or calorie intake [24–26].

In this study, we attempted to investigate the relationship between renal dysfunction and cognitive decline, and which cognitive function domains are significantly related to renal function in community-dwelling older adults enrolled in KFACS.

## Methods

### Study population and design

This cross-sectional analysis was based on data from the KFACS. Of the 3014 participants who were enrolled in the KFACS during the first and second years (2016–2017), those without demographic information or baseline laboratory data (*n* = 6) and those who had cerebrovascular disease or dementia in their medical history (*n* = 161) were excluded. A total of 2847 participants (1333 men and 1514 women) were enrolled as the final analysis sample. All study procedures complied with the ethical guidelines of the 1975 Declaration of Helsinki, as revised in 2000.

The KFACS protocol was approved by the Institutional Review Boards (IRBs) of the Clinical Research Ethics Committee of each center, and the approval number from Kyung Hee University Medical Center, the coordinating center, was 2015–12-103. Written informed consents were obtained from all participants or their legal guardians after providing them with sufficient explanation. This study was exempt from review by the IRB of Kyung Hee University Medical Center (IRB number: 2019–09-047).

### Cognitive function test

To evaluate global cognitive function, we used the Mini-mental State Examination-Korean version (MMSE), which is a screening tool for cognitive decline. Global cognitive impairment was defined as a score of > 1.5 standard deviation (SD) below the mean of the age-, sex-, and education level-matched Korean norms on MMSE (Supplementary Table 1) [27]. We also conducted neuropsychological tests included in the Consortium to Establish a Registry for Alzheimer’s Disease-Korean version (CERAD) assessment battery [28] and Frontal Assessment Battery (FAB) [29]. The CERAD neuropsychological assessment battery is a comprehensive cognitive function assessment tool and consists of eight tests (verbal fluency, Boston naming test, MMSE, word list memory, constructional praxis, word list recall, word list recognition, and constructional praxis recall). In this study, we used the word list memory, word list recall, and word list recognition tests for the immediate, recent memory, and recognition function domains. Digit span forward and digit span backward were used to test the attention-concentration and working memory domains, and the Trail Making Test (TMT) A was used for assessing the processing speed. Further, the FAB was used to test the executive function domain.

### Other variables and definitions

Baseline variables including age; sex; education level; smoking and alcohol consumption; Geriatric Depression Scale-Korean version (GDS) with 15 items [30]; and underlying medical problems such as hypertension, diabetes mellitus, dyslipidemia, and coronary artery disease were investigated. Body mass index (BMI) was calculated as weight divided by the square of height (kg/m^2^). Appendicular skeletal muscle mass (ASM) was measured using DEXA (Hologic DEXA; Hologic Inc., Bedford, MA, USA, and Lunar; GE Healthcare, Madison, WI, USA). ASM index was calculated as the sum of the lean mass from both arms and legs (kg) divided by the square of height (m^2^). Further, eGFR (mL/min/1.73m^2^) was calculated using the CKD Epidemiology Collaboration equation [31]. Proteinuria was defined as a score of ≥1+ on a dipstick test in a random urine sample.

### Statistical analysis

Continuous variables are presented as mean ± SD values, and categorical data are reported as frequencies and percentages. Analysis of variance was used for normally distributed continuous data, the Kruskal-Wallis test was used for non-normally distributed continuous data, with Bonferroni post hoc analysis. Chi-squared test was used for categorical data. Spearman’s analyses were used to evaluate the correlation between eGFR and neuropsychological tests. The correlations between zero-score standardized neuropsychological function tests and relevant variables were investigated by linear regression analyses, and sex-stratified analysis was also performed. In multivariate models included significantly associated parameters according to their weight in univariate analysis. Further, the relationship between eGFR and global cognitive impairment was investigated using logistic regression analysis, and the possible confounders including smoking history, alcohol consumption, BMI, GDS, albumin, low-density lipoprotein, hemoglobin, proteinuria, and ASM index were used for adjustment. Log-transformed values of eGFR were used in linear regression, and quartiles of eGFR were used in logistic regression analysis because of a skewed distribution. Unadjusted and adjusted odds ratios (ORs) with 95% confidence intervals (CIs) were obtained, and a *P* value of < 0.05 was considered statistically significant. All statistical analyses were performed using SPSS software version 20.0 (SPSS Inc., Chicago, IL, USA).

## Results

### Study population characteristics

The baseline characteristics of all participants in this study are shown in Table 1. The mean age was 76.0 ± 3.9 years and 1333 (46.8%) were men. The mean eGFR (all in mL/min/1.73 m^2^) was 77.5 ± 14.3 and the mean education duration was 8.4 ± 5.1 years. Of the participants, 1637 (57.5%) had hypertension, 614 (21.6%) had diabetes mellitus, and 222 (8.0%) had coronary artery disease.

**Table 1:** Demographic baseline characteristics of the study group according to renal function

	Overall (*N* = 2847)	Quartile 1 (*n* = 711)	Quartile 2 (*n* = 712)	Quartile 3 (*n* = 712)	Quartile 4 (*n* = 712)	*P*
eGFR, mL/min/1.73 m^2^	77.5 ± 14.3	91.7 ± 3.2	84.9 ± 1.8^a,c^	76.1 ± 3.7^a,b^	57.2 ± 10.8^a,b,c^	< 0.001
**Demographic characteristics**						
Age, years	76.0 ± 3.9	73.7 ± 3.1	76.2 ± 3.6^a,c^	76.7 ± 4.0^a,b^	77.4 ± 3.8^a,b,c^	< 0.001
Men, n (%)	1333 (46.8)	262 (36.8)	318 (44.7)^a^	361 (50.7)^a^	392 (55.1)^a,b^	< 0.001
Education, years	8.4 ± 5.1	8.0 ± 4.8	8.2 ± 5.0	8.8 ± 5.1^a^	8.7 ± 5.4^a^	0.005
Smoking, n (%)	182 (6.4)	34 (4.8)	52 (7.3)	44 (6.2)	52 (7.3)	0.164
Alcohol consumption, n (%)	1416 (49.7)	360 (50.6)	363 (51.0)	364 (51.1)	367 (51.5)	0.717
**Medical history**						
Hypertension, n (%)	1637 (57.5)	335 (47.2)	379 (53.2)	430 (60.4)^a,b^	493 (69.2)^a,b,c^	< 0.001
Diabetes mellitus, n (%)	614 (21.6)	135 (19.0)	135 (19.0)	138 (19.4)	206 (29.0)^a,b,c^	< 0.001
Dyslipidemia, n (%)	898 (31.9)	225 (32.1)	218 (30.8)	217 (31.0)	238 (33.7)	0.629
Coronary artery disease, n (%)	222 (8.0)	35 (5.0)	54 (7.7)	54 (7.7)	79 (11.2)^a^	< 0.001
**Clinical results**						
BMI, kg/m^2^	24.4 ± 3.1	24.1 ± 3.0	24.3 ± 2.9	24.3 ± 3.0	24.8 ± 3.2^a,b^	0.001
ASM index, kg/m^2^	6.6 ± 1.1	6.4 ± 1.1	6.6 ± 1.1	6.6 ± 1.2^a^	6.7 ± 1.2^a,b^	< 0.001
SBP, mmHg	131.1 ± 15.7	130.5 ± 15.4	131.2 ± 15.3	131.4 ± 15.9	131.3 ± 16.1	0.703
DBP, mmHg	77.4 ± 9.3	78.0 ± 9.1	77.4 ± 9.1	77.5 ± 9.4	76.8 ± 9.8	0.137
GDS score	3.2 ± 3.7	3.1 ± 3.6	3.3 ± 3.7	2.9 ± 3.5	3.5 ± 3.8	0.061
**Laboratory results**						
Albumin, g/dL	4.2 ± 0.4	4.16 ± 0.4	4.17 ± 0.4	4.24 ± 0.4	4.20 ± 0.4	0.001
Creatinine, mg/dL	0.8 ± 0.3	0.6 ± 0.1	0.7 ± 0.1^a,c^	0.9 ± 0.1^a,b^	1.2 ± 0.4^a,b,c^	< 0.001
Triglyceride, mg/dL	121.8 ± 60.9	114.7 ± 54.1	115.5 ± 55.5	123.3 ± 65.8	133.5 ± 65.7^a,b,c^	< 0.001
HDL-C, mg/dL	52.4 ± 13.9	54.4 ± 14.0	53.4 ± 13.6	52.3 ± 13.8^a^	49.5 ± 13.9^a,b,c^	< 0.001
LDL-C, mg/dL	108.5 ± 33.3	111.2 ± 31.3	109.1 ± 33.9	108.8 ± 33.7	104.9 ± 33.9^a^	0.004
Sodium, mmol/L	141.3 ± 2.4	141.1 ± 2.2	141.4 ± 2.3	141.5 ± 2.7	141.0 ± 2.4^b,c^	0.003
HbA1c, %	6.10 ± 0.83	6.02 ± 0.792	6.02 ± 0.762	6.01 ± 0.834	6.19 ± 0.909^a,b,c^	< 0.001
Hemoglobin, g/dL	13.4 ± 1.4	13.4 ± 1.3	13.5 ± 1.4	13.6 ± 1.4	13.2 ± 1.6^b,c^	< 0.001
hsCRP, mg/dL	1.5 ± 2.6	1.3 ± 2.2	1.5 ± 2.8	1.5 ± 2.5	1.8 ± 2.9^a,b,c^	< 0.001
Proteinuria, n (%)	84 (3.0)	7 (8.3)	8 (9.5)	17 (20.2)	52 (61.9)^a,b,c^	< 0.001

*ASM* appendicular skeletal muscle mass, *BMI* body mass index, *DBP* diastolic blood pressure, *eGFR* estimated glomerular filtration rate, *GDS* Geriatric Depression Scale-Korean version, *HbA1c* glycated hemoglobin, *HDL-C* high-density lipoprotein, *hsCRP* high-sensitivity C-reactive protein, *LDL-C* low-density lipoprotein, *SBP* systolic blood pressure

^a^*p* < 0.05 vs. quartile 1

^b^*p* < 0.05 vs. quartile 2

^c^*p* < 0.05 vs. quartile 3

The distribution of participants in each eGFR quartile are also shown in Table 1. The mean eGFR was 91.7 ± 3.2 in quartile 1, 84.9 ± 1.8 in quartile 2, 76.1 ± 3.7 in quartile 3, and 57.2 ± 10.8 in quartile 4. Participants with lower renal function were older and included more men. They also had higher muscle mass; higher level of triglyceride and high-sensitivity C-reactive protein (hsCRP); lower levels of low-density lipoprotein cholesterol; and higher proportions of patients with hypertension, diabetes mellitus, coronary artery disease, and proteinuria.

The baseline characteristics in terms of cognitive function are shown in Table 2. Generally, participants with lower eGFR had lower cognitive function scores than participant with higher eGFR in MMSE, word list memory, word list recall, and word list recognition. Higher test scores indicate higher cognitive function, except for TMT A in which lower scores are better.

**Table 2:** Cognitive baseline characteristics of the study group according to renal function

	Overall (*N* = 2847)	Quartile 1 (*n* = 711)	Quartile 2 (*n* = 712)	Quartile 3 (*n* = 712)	Quartile 4 (*n* = 712)	*P*
MMSE	25.5 ± 3.3	25.8 ± 3.2	25.3 ± 3.4^a^	25.6 ± 3.3	25.4 ± 3.5^a^	0.013
Word list memory	16.6 ± 4.3	17.6 ± 4.3	16.7 ± 4.4^a^	16.4 ± 4.2^a^	15.9 ± 4.3^a,b^	< 0.001
Word list recall	5.5 ± 2.1	5.9 ± 2.0	5.6 ± 2.1^a^	5.4 ± 2.1^a^	5.1 ± 2.1^a,b^	< 0.001
Word list recognition	8.6 ± 1.9	8.8 ± 1.8	8.6 ± 1.9	8.5 ± 1.9	8.3 ± 2.0^a^	< 0.001
Digit span forward	6.6 ± 2.6	6.6 ± 2.6	6.5 ± 2.7	6.6 ± 2.6	6.5 ± 2.7	0.461
Digit span backward	4.0 ± 1.8	4.1 ± 1.8	3.9 ± 1.8	4.1 ± 1.8	4.0 ± 1.8	0.133
TMT A, sec	84.3 ± 63.9	79.9 ± 57.2	85.4 ± 63.9	83.8 ± 67.1	88.1 ± 66.6	0.060
FAB (range: 0–18)	13.4 ± 3.1	13.5 ± 3.0	13.3 ± 3.1	13.6 ± 2.9	13.3 ± 3.3	0.104

*MMSE* Mini-mental State Examination-Korean version, *TMT A* Trail Making Test A, *FAB* Frontal Assessment Battery

^a^*p* < 0.05 vs. quartile 1

^b^*p* < 0.05 vs. quartile 2

^c^*p* < 0.05 vs. quartile 3

### Association between renal function and cognitive function domains

The association between neuropsychological function tests and eGFR were investigated using Spearman’s correlation analyses (Table 3). The result showed positive correlation between eGFR and MMSE, word list memory, word list recall, and word list recognition; and negative correlation between eGFR and TMT A. Linear regression analysis was performed for word list memory, word list recall, and word list recognition (Tables 4, 5 and 6), which showed statistically significant correlation with eGFR and also significant differences between eGFR quartiles (Table 2).

**Table 3:** Univariable Spearman’s correlation coefficients

	eGFR, mL/min/1.73m^2^	
	rho	*P*
MMSE	0.041	0.030
Word list memory	0.141	< 0.001
Word list recall	0.150	< 0.001
Word list recognition	0.092	< 0.001
Digit span forward	0.026	0.160
Digit span backward	0.019	0.321
TMT A	−0.040	0.034
FAB	0.011	0.564

*MMSE* Mini-mental State Examination-Korean version, *TMT A* Trail Making Test A, *FAB* Frontal Assessment Battery

**Table 4:** Simple and multiple linear regression analyses of word list memory score

	Simple			Multiple		
	estimate	95% CI	*P*	estimate	95% CI	*P*
log eGFR	1.31	0.94, 1.67	< 0.001	0.53	0.16, 0.90	0.005
Age, years	− 0.08	− 0.09, − 0.07	< 0.001	− 0.06	− 0.07, − 0.05	< 0.001
Men, *n* (%)	− 0.10	− 0.17, − 0.02	0.010	− 0.30	− 0.39, − 0.21	< 0.001
Education, years	0.06	0.06, 0.07	< 0.001	0.06	0.05, 0.07	< 0.001
Smoking, *n* (%)	−0.26	− 0.41, − 0.10	0.001	− 0.07	− 0.22, 0.07	0.334
Alcohol consumption, *n* (%)	0.08	0.00, 0.15	0.040	0.02	−0.05, 0.09	0.570
BMI, kg/m^2^	0.02	0.01, 0.04	< 0.001	0.02	0.01, 0.03	0.002
GDS score	−0.05	− 0.06, − 0.04	< 0.001	− 0.02	− 0.03, − 0.01	< 0.001
Albumin, g/dL	0.41	0.26, 0.55	< 0.001	0.17	0.03, 0.30	0.015
LDL-C, mg/dL	0.00	0.00, 0.00	0.939	NS	.	.
Sodium, mmol/L	0.03	0.01, 0.04	< 0.001	0.01	0.00, 0.03	0.089
HbA1c, %	0.00	−0.05, 0.05	0.972	NS	.	.
Hemoglobin, g/dL	0.04	0.02, 0.07	0.001	0.01	−0.02, 0.04	0.552
hsCRP, mg/dL	−0.02	−0.03, − 0.01	0.004	0.00	− 0.01, 0.02	0.676
ASM index, kg/m^2^	−0.01	−0.04, 0.02	0.530	NS	.	.
Proteinuria, *n* (%)	−0.28	− 0.50, − 0.07	0.010	−0.08	− 0.28, 0.12	0.455

*ASM* appendicular skeletal muscle mass, *BMI* body mass index, *eGFR* estimated glomerular filtration rate, *GDS* Geriatric Depression Scale-Korean version, *HbA1c* glycated hemoglobin, *hsCRP* high-sensitivity C-reactive protein, *LDL-C* low-density lipoprotein cholesterol

**Table 5:** Simple and multiple linear regression analyses of word list recall scores

	Simple			Multiple		
	estimate	95% CI	*P*	estimate	95% CI	*P*
log eGFR	1.47	1.11, 1.84	< 0.001	0.86	0.49, 1.23	< 0.001
Age, years	−0.08	− 0.08, − 0.07	< 0.001	−0.06	− 0.07, − 0.05	< 0.001
Men, *n* (%)	0.01	− 0.07, 0.08	0.877	NS	.	.
Education, years	0.05	0.05, 0.06	< 0.001	0.04	0.04, 0.05	< 0.001
Smoking, *n* (%)	−0.12	− 0.28, 0.04	0.133	NS	.	.
Alcohol consumption, *n* (%)	0.16	0.08, 0.23	< 0.001	0.06	−0.01, 0.13	0.106
BMI, kg/m^2^	0.02	0.01, 0.03	< 0.001	0.02	0.01, 0.03	< 0.001
GDS score	−0.05	− 0.06, − 0.04	< 0.001	− 0.02	− 0.03, − 0.01	< 0.001
Albumin, g/dL	0.33	0.19, 0.48	< 0.001	0.13	0.00, 0.27	0.057
LDL-C, mg/dL	0.00	0.00, 0.00	0.116	NS	.	.
Sodium, mmol/L	0.02	0.01, 0.04	0.008	0.01	0.00, 0.03	0.045
HbA1c, %	−0.03	− 0.08, 0.01	0.144	NS	.	.
Hemoglobin, g/dL	0.06	0.03, 0.09	< 0.001	−0.02	−0.04, 0.01	0.250
hsCRP, mg/dL	−0.02	−0.03, 0.00	0.021	0.00	−0.01, 0.02	0.664
ASM index, kg/m^2^	0.02	−0.01, 0.05	0.201	NS	.	.
Proteinuria, *n* (%)	−0.38	− 0.59, − 0.16	< 0.001	−0.20	− 0.40, 0.01	0.061

*ASM* appendicular skeletal muscle mass, *BMI* body mass index, *eGFR* estimated glomerular filtration rate, *GDS* Geriatric Depression Scale-Korean version, *HbA1c* glycated hemoglobin, *hsCRP* high-sensitivity C-reactive protein, *LDL-C* low-density lipoprotein cholesterol

**Table 6:** Simple and multiple linear regression analyses of word list recognition scores

	Simple			Multiple		
	estimate	95% CI	*P*	estimate	95% CI	*P*
log eGFR	0.87	0.51, 1.24	< 0.001	0.43	0.04, 0.82	0.030
Age, years	−0.05	− 0.05, − 0.04	< 0.001	−0.03	− 0.04, − 0.02	< 0.001
Men, *n* (%)	0.02	− 0.06, 0.09	0.635	NS	.	.
Education, years	0.03	0.02, 0.04	< 0.001	0.03	0.02, 0.03	< 0.001
Smoking, *n* (%)	−0.11	−0.27, 0.05	0.169	NS	.	.
Alcohol consumption, *n* (%)	0.06	−0.01, 0.14	0.087	0.00	−0.07, 0.08	0.968
BMI, kg/m^2^	0.01	0.00, 0.03	0.022	0.01	0.00, 0.03	0.026
GDS score	−0.03	−0.04, − 0.02	< 0.001	− 0.01	−0.02, − 0.00	0.114
Albumin, g/dL	0.12	−0.02, 0.26	0.105	NS	.	.
LDL-C, mg/dL	0.00	0.00, 0.00	0.605	NS	.	.
Sodium, mmol/L	0.01	0.00, 0.03	0.056	0.01	0.00, 0.03	0.157
HbA1c, %	0.00	−0.05, 0.04	0.926	NS	.	.
Hemoglobin, g/dL	0.04	0.01, 0.07	0.002	0.00	−0.03, 0.03	0.970
hsCRP, mg/dL	−0.02	−0.03, 0.00	0.013	−0.01	− 0.02, 0.01	0.326
ASM index, kg/m^2^	0.02	−0.01, 0.05	0.230	NS	.	.
Proteinuria, *n* (%)	−0.33	− 0.55, − 0.11	0.003	−0.23	− 0.45, − 0.02	0.035

*ASM* appendicular skeletal muscle mass, *BMI* body mass index, *eGFR* estimated glomerular filtration rate, *GDS* Geriatric Depression Scale-Korean version, *HbA1c* glycated hemoglobin, *hsCRP* high-sensitivity C-reactive protein, *LDL-C* low-density lipoprotein cholesterol

In simple linear regression analysis, word list memory score was positively correlated with log eGFR (β = 1.31, *P* < 0.001), education level, alcohol consumption, BMI, albumin, plasma sodium level, and hemoglobin concentration; and negatively correlated with age, male sex, smoking, GDS score, hsCRP, and proteinuria. After multiple linear regression analysis, word list memory score was found to correlate significantly with log eGFR (β = 0.53, *P* = 0.005) (Table 4).

In word list recall, log eGFR (β = 1.47, *P* < 0.001), education level, alcohol consumption, BMI, albumin, plasma sodium level, and hemoglobin concentration showed positive correlation; and age, GDS score, hsCRP, and proteinuria showed negative correlation. After adjustment of relevant parameters, the positive correlation between word list recall score and log eGFR (β = 0.86, *P* < 0.001) remained significant (Table 5).

Table 6 shows the linear regression analyses of word list recognition. In simple linear regression, word list recognition score was positively correlated with log eGFR (β = 0.87, *P* < 0.001), education level, BMI, and hemoglobin concentration; and negatively correlated with age, GDS score, hsCRP, and proteinuria. After adjustment of confounding variables, the positive correlation between word list recognition score and log eGFR (β = 0.43, *P* = 0.030) remained significant.

Sex-stratified linear regression analysis of word list memory and word list recall showed significant association with log eGFR in women. Word list memory was positively correlated with log eGFR (β = 0.75, *P* = 0.005) (Supplementary Table 2), and word list recall was also positively correlated with log eGFR (β = 0.87, *P* = 0.002) (Supplementary Table 3). However, in men, word list memory (β = 0.25, *P* = 0.324) and word list recall (β = 0.51, *P* = 0.051) showed positive correlations with eGFR which were not statistically significant (Supplementary Tables 4 and 5). In word list recognition, sex-stratified analysis did not show statistically significant association with log eGFR in both men and women (table not shown).

### Association between renal function and cognitive impairment

Spearman’s correlation analyses showed positive correlation between eGFR and MMSE (Table 3). However, the association between MMSE and eGFR was not linear (Supplementary Table 6). Table 7 shows the association between eGFR quartiles and global cognitive impairment. In the crude model, the association with global cognitive impairment was found in quartile 2 (unadjusted OR: 1.547, 95% CI: 1.130–2.118, *P* = 0.006) than in quartile 1. The ORs of global cognitive impairment were 1.484 (95% CI: 1.082–2.035, *P* = 0.014) in quartile 3 and 1.465 (95% CI: 1.067–2.010, *P* = 0.018) in quartile 4 compared with quartile 1. After controlling for potential confounding variables, the association with global cognitive impairment in quartile 2 was stronger (adjusted OR: 1.535, 95% CI: 1.111–2.120, *P* = 0.009). Moreover, the ORs of global cognitive impairment were 1.501 (95% CI: 1.084–2.079, *P* = 0.014) in quartile 3 and 1.423 (95% CI: 1.022–1.983, *P* = 0.037) in quartile 4.

**Table 7:** Logistic regression analysis of the association between estimated glomerular filtration rate quartiles and global cognitive impairment

Variable	No. of patients (%)	Unadjusted		Adjusted^a^	
		OR (95% CI)	*P*	OR (95% CI)	*P*
Quartile 1	77 (10.8)	1.00		1.00	
Quartile 2	113 (15.9)	1.547 (1.130–2.118)	0.006	1.535 (1.111–2.120)	0.009
Quartile 3	108 (15.2)	1.484 (1.082–2.035)	0.014	1.501 (1.084–2.079)	0.014
Quartile 4	106 (14.9)	1.465 (1.067–2.010)	0.018	1.423 (1.022–1.983)	0.037

^a^Adjusted for smoking, alcohol consumption, body mass index, Geriatric Depression Scale-Korean version score, albumin, low-density lipoprotein cholesterol, hemoglobin, proteinuria, and appendicular muscle mass index

## Discussion

In this cross-sectional study, we showed the association between renal function and neurocognitive function in older adults. The main results were as follows: (1) the immediate, recent memory, and recognition function domains were independently associated with eGFR, (2) particularly in women, the memory domains were significantly associated with eGFR, and (3) mild decline of renal function was also strongly associated with global cognitive impairment.

Previous studies have identified the association between renal function and many domains of cognitive function, but they have presented various results. In post-menopausal women, CKD was reported to increase the risk of dysfunction in the global cognition, executive function, language and memory domains [5]. Moreover, in a study in Japanese elderly people, lower levels of eGFR were independently associated with lower cognitive performance in attention and processing speed [10]. In this study, immediate, recent memory, and recognition function domains were associated with renal function in the general elderly population. Further, the immediate and recent memory domains, showed strong association with renal function mainly in women. Among the previous studies that evaluated the association between renal dysfunction and cognitive decline, sex differences were observed in some studies. Mild renal dysfunction has found to be associated with faster cognitive decline in women [32]. Jassal et al. showed albuminuria is associated with greater decline of cognitive function in older adults, particularly in men [33]. However, to our knowledge, the reasons of sex differences in the association between renal dysfunction and cognitive function have not been studied previously. Thus, further investigation might be needed to elucidate the mechanism of sex differences of cognitive decline in patients with renal dysfunction.

Furthermore, these memory domains shared common independent determinants including, eGFR, age, education level, BMI, and GDS. Which means, memory domains can be affected by not only eGFR, but also educational level, emotional and nutritional status, which had been demonstrated before as a risk factor of cognitive dysfunction [25, 28, 34, 35]. Soysal et al. showed that the geriatric ESRD patients had lower cognitive function, and it might be affected not only their renal dysfunction, but also complex geriatric conditions [36]. However, after adjustment of these confounding factors, memory domains showed independent association with eGFR. By comparison with the memory domains, recognition function showed no differences between men and women, and less affected by emotional status.

Moderate to severe renal dysfunction has been recognized as a risk factor of cognitive decline. However, we showed mild decline of renal function was also associated with global cognitive impairment. There might be several mechanisms that explain the association between mild renal dysfunction and global cognitive impairment. One possible explanation is a vascular mechanism including endothelial dysfunction [37–39]. In former studies, minor renal dysfunction has been established as an independent risk factor for cardiovascular disease [40, 41]. Similar vascular risk factors were reported to affect both the cardiovascular and cerebrovascular systems, and many of them overlapped with risk factors for kidney disease, including smoking, diabetes mellitus, and hypertension [13, 14, 42–44].

This study had several strengths. First, this study was performed with a large cohort comprised of community-dwelling participants nationwide, not clinic-based participants. Thereby, the results of this study could possibly be applied to the general elderly population. Second, cognitive function was assessed using not only MMSE but also CERAD and FAB, thus allowing us to investigate the comprehensive cognitive function. Furthermore, we defined global cognitive impairment as a score of > 1.5 SD below the mean of MMSE with consideration of age, sex, and education level, which enabled us to more precisely grade the cognitive function of the participants [27]. Third, in the elderly population, cognitive function decline is affected not only by aging but also by general medical condition, muscle strength, nutritional status, and emotional status [10, 24–26]. Therefore, it is necessary to determine if the relationship between renal dysfunction and cognitive decline still exists after adjustment for these confounding factors. We considered various laboratory data and medical histories, including proteinuria, muscle mass and emotional status of older adults based on GDS scores, which are known confounding factors.

However, this study also had several limitations. First, we performed only one measurement of serum creatinine and calculated the eGFR, using CKD-EPI equation. Thus, overestimation or underestimation of renal function and misclassification could exist. Also, as the original CKD-EPI equation was developed and validated based on large population composed of Caucasians and African-Americans, and relatively few participants were Asian [31], validation of the equation estimating GFR might be needed. In Korea, the original CKD-EPI equation was valid for Korean population [45]. Moreover, the Korean Society of Nephrology recommends using the original CKD-EPI equations without ethnic adjustment for estimating GFR [46]. But clear conclusion about this ethnic coefficient in Korean may need further investigation. Second, because we used a community-based cohort, the renal function of the participants was not evenly distributed. The proportion of participants with severe renal dysfunction was relatively low, with 12.6% (*n* = 360) with baseline eGFR < 60 mL/min/1.73 m^2^ and 0.8% (*n* = 25) with eGFR < 30 mL/min/1.73 m^2^ (Supplementary Table 7). This may be explained by the fact that the study sample was composed of volunteers who may have higher cognitive function or may be in better general condition. Thus, we had to divide the study population with eGFR quartiles, not by CKD stages. Also, a potential underestimation of cognitive impairment in this category might be present, and we could not show a dose-dependent relationship between renal dysfunction and global cognitive impairment. Third, this study was cross-sectional, which did not allow for us to determine causal associations between renal dysfunction and cognitive impairment. Fourth, in elderly population, CKD increases the chance of taking multiple medications, electrolytes imbalances, and infections. Moreover, it might increase the risk of delirium, which could affect the cognitive function temporarily. However, our data had limited information about delirium of the participants, the effect of delirium on cognitive function in older adults could not be considered. Finally, we used observational data, and even though most of the important confounders were identified and included, unchecked confounders might exist.

## Conclusions

In summary, in the general elderly population, the immediate, recent memory, and recognition domains were significantly related to renal function, and even a mild decline of renal function was independently associated with global cognitive impairment. These results suggest that the early stages of renal dysfunction could be an effective target to prevent worsening of cognitive impairment. Therefore, regular monitoring and early detection of mild renal dysfunction in elderly population might be needed. Further longitudinal studies are needed to ascertain the underlying mechanisms and causal relationships between renal function and cognitive function in older adults.

## Supplementary Information

**Additional file 1:** **Table S1.** Cut-points for global cognitive impairment by age-, sex-, education- level matched norms of MMSE-KC. **Table S2.** Simple and multiple linear regression analyses of word list memory scores in women. **Table S3.** Simple and multiple linear regression analyses of word list recall scores in women. **Table S4.** Simple and multiple linear regression analyses of word list memory scores in men. **Table S5.** Simple and multiple linear regression analyses of word list recall scores in men. **Table S6.** Simple and multiple linear regression analysis of MMSE. **Table S7.** Logistic regression analysis of the association between CKD stages and global cognitive impairment.

## Data Availability

The datasets used and/or analyzed during the current study are available from the corresponding author on reasonable request.

## Abbreviations

ASM: Appendicular skeletal muscle mass
BMI: Body mass index
CERAD: Consortium to Establish a Registry for Alzheimer’s Disease-Korean version
CI: Confidence interval
CKD: Chronic kidney disease
DBP: Diastolic blood pressure
eGFR: Estimated glomerular filtration rate
ESRD: End-stage renal disease
FAB: Frontal Assessment Battery
GDS: Geriatric Depression Scale-Korean version
HbA1c: Glycated hemoglobin
HDL-C: High-density lipoprotein cholesterol
hsCRP: High-sensitivity C-reactive protein
KFACS: Korean Frailty and Aging Cohort Study
LDL-C: Low-density lipoprotein cholesterol
MMSE: Mini-mental State Examination-Korean version
OR: Odds ratio
SBP: Systolic blood pressure
TMT A: Trail Making Test A
