# Supplementary material for: Renal dysfunction is associated with decline of cognitive function in community-dwelling older adults: Korean frailty and aging cohort study
Source: BMC Geriatr. 2020 Nov 10;20:462. doi: 10.1186/s12877-020-01862-8 (PMC7654158; doi:10.1186/s12877-020-01862-8)
Supplement: Supplementary file 1 — Additional file 1: Table S1. Cut-points for global cognitive impairment by age-, sex-, education- level matched norms of MMSE-KC. Table S2. Simple and multiple linear regression analyses of word list memory scores in women. Table S3. Simple and multiple linear regression analyses of word list recall scores in women. Table S4. Simple and multiple linear regression analyses of word list memory scores in men. Table S5. Simple and multiple linear regression analyses of word list recall scores in men. Table S6. Simple and multiple linear regression analysis of MMSE. Table S7. Logistic regression analysis of the association between CKD stages and global cognitive impairment. [file 12877_2020_1862_MOESM1_ESM.docx]

**Supplementary Table 1 Cut-points for global cognitive impairment by age-, sex-, education- level matched norms of MMSE-KC**

|  | Education (years) | | | | | |
| --- | --- | --- | --- | --- | --- | --- |
|  | 0-3 | | 4-6 | | ≥7 | |
| Age (years) | Men | Women | Men | Women | Men | Women |
| 70-74 | 19.7 | 16.7 | 23.3 | 22.0 | 25.5 | 24.9 |
| 75-79 | 19.7 | 15.1 | 22.3 | 21.6 | 25.4 | 23.9 |
| 80-90 | 17.3 | 13.5 | 21.2 | 20.4 | 24.8 | 22.9 |

**Supplementary Table 2 Simple and multiple linear regression analyses of word list memory scores in women**

|  | Simple | | |  | Multiple | | | |
| --- | --- | --- | --- | --- | --- | --- | --- | --- |
|  | estimate | 95% CI | *P* |  | estimate | 95% CI | *P* |  |
| log eGFR | 2.06 | 1.49, 2.64 | <0.001 |  | 0.75 | 0.22, 1.27 | 0.005 |  |
| Age, years | -0.08 | -0.10, -0.07 | <0.001 |  | -0.05 | -0.06, -0.04 | <0.001 |  |
| Men, *n* (%) | NS | . | . |  | NS | . | . |  |
| Education, years | 0.09 | 0.08, 0.10 | <0.001 |  | 0.07 | 0.06, 0.08 | <0.001 |  |
| Smoking, *n* (%) | -0.51 | -1.06, 0.04 | 0.069 |  | 0.09 | -0.37, 0.55 | 0.708 |  |
| Alcohol consumption, *n* (%) | 0.07 | -0.04, 0.18 | 0.184 |  | NS | . | . |  |
| BMI, kg/m^2^ | 0.01 | -0.01, 0.03 | 0.267 |  | NS | . | . |  |
| GDS score | -0.06 | -0.07, -0.05 | <0.001 |  | -0.02 | -0.03, -0.01 | <0.001 |  |
| Albumin, g/dL | 0.45 | 0.24, 0.66 | <0.001 |  | 0.19 | 0.00, 0.38 | 0.045 |  |
| LDL-C, mg/dL | 0.00 | 0.00, 0.00 | 0.562 |  | NS | . | . |  |
| Sodium, mmol/L | 0.04 | 0.02, 0.06 | 0.001 |  | 0.02 | 0.00, 0.04 | 0.033 |  |
| HbA1c, % | 0.00 | -0.07, 0.07 | 0.991 |  | NS | . | . |  |
| Hemoglobin, g/dL | 0.11 | 0.06, 0.15 | <0.001 |  | 0.02 | -0.03, 0.06 | 0.444 |  |
| hsCRP, mg/dL | 0.00 | -0.03, 0.02 | 0.690 |  | NS | . | . |  |
| ASM index, kg/m^2^ | 0.01 | -0.05, 0.06 | 0.764 |  | NS | . | . |  |
| Proteinuria, *n* (%) | -0.23 | -0.66, 0.20 | 0.298 |  | NS | . | . |  |

ASM, appendicular skeletal muscle mass; BMI, body mass index; eGFR, estimated glomerular filtration rate; GDS, Geriatric Depression Scale-Korean version; HbA1c, glycated hemoglobin; hsCRP, high-sensitivity C-reactive protein; LDL-C, low-density lipoprotein cholesterol.

**Supplementary Table 3 Simple and multiple linear regression analyses of word list recall scores in women**

|  | Simple | | |  | Multiple | | | |
| --- | --- | --- | --- | --- | --- | --- | --- | --- |
|  | estimate | 95% CI | *P* |  | estimate | 95% CI | *P* |  |
| log eGFR | 2.09 | 1.52, 2.65 | <0.001 |  | 0.87 | 0.32, 1.41 | 0.002 |  |
| Age, years | -0.08 | -0.09, -0.07 | <0.001 |  | -0.05 | -0.06, -0.04 | <0.001 |  |
| Men, *n* (%) | NS | . | . |  | NS | . | . |  |
| Education, years | 0.08 | 0.07, 0.09 | <0.001 |  | 0.06 | 0.05, 0.07 | <0.001 |  |
| Smoking, *n* (%) | -0.56 | -1.11, -0.03 | 0.038 |  | -0.05 | -0.53, 0.44 | 0.854 |  |
| Alcohol consumption, *n* (%) | 0.12 | 0.01, 0.22 | 0.035 |  | 0.05 | -0.04, 0.15 | 0.275 |  |
| BMI, kg/m^2^ | 0.00 | -0.02, 0.02 | 0.936 |  | NS | . | . |  |
| GDS score | -0.05 | -0.06, -0.03 | <0.001 |  | -0.01 | -0.03, 0.00 | 0.023 |  |
| Albumin, g/dL | 0.31 | 0.10, 0.52 | 0.004 |  | 0.10 | -0.09, 0.30 | 0.309 |  |
| LDL-C, mg/dL | 0.00 | 0.00, 0.00 | 0.793 |  | NS | . | . |  |
| Sodium, mmol/L | 0.03 | 0.01, 0.05 | 0.006 |  | 0.02 | 0.00, 0.04 | 0.093 |  |
| HbA1c, % | -0.04 | -0.10, 0.03 | 0.272 |  | NS | . | . |  |
| Hemoglobin, g/dL | 0.08 | 0.04, 0.13 | <0.001 |  | 0.01 | -0.04, 0.05 | 0.808 |  |
| hsCRP, mg/dL | 0.01 | -0.02, 0.03 | 0.564 |  | NS | . | . |  |
| ASM index, kg/m^2^ | 0.01 | -0.04, 0.07 | 0.646 |  | NS | . | . |  |
| Proteinuria, *n* (%) | -0.48 | -0.90, -0.06 | 0.025 |  | -0.23 | -0.61, 0.15 | 0.233 |  |

ASM, appendicular skeletal muscle mass; BMI, body mass index; eGFR, estimated glomerular filtration rate; GDS, Geriatric Depression Scale-Korean version; HbA1c, glycated hemoglobin; hsCRP, high-sensitivity C-reactive protein; LDL-C, low-density lipoprotein cholesterol.

**Supplementary Table 4 Simple and multiple linear regression analyses of word list memory scores in men**

|  | Simple | | |  | Multiple | | | |
| --- | --- | --- | --- | --- | --- | --- | --- | --- |
|  | estimate | 95% CI | *P* |  | estimate | 95% CI | *P* |  |
| log eGFR | 0.64 | 0.17, 1.11 | 0.007 |  | 0.25 | -0.25, 0.75 | 0.324 |  |
| Age, years | -0.07 | -0.09, -0.06 | <0.001 |  | -0.07 | -0.08, -0.05 | <0.001 |  |
| Men, *n* (%) | NS | . | . |  | NS | . | . |  |
| Education, years | 0.06 | 0.05, 0.07 | <.0001 |  | 0.05 | 0.04, 0.06 | <0.001 |  |
| Smoking, *n* (%) | -0.20 | -0.36, -0.04 | 0.016 |  | -0.11 | -0.26, 0.05 | 0.174 |  |
| Alcohol consumption, *n* (%) | 0.16 | 0.05, 0.26 | 0.003 |  | 0.07 | -0.03, 0.18 | 0.164 |  |
| BMI, kg/m^2^ | 0.04 | 0.02, 0.06 | <0.001 |  | 0.02 | 0.01, 0.04 | 0.006 |  |
| GDS score | -0.06 | -0.07, -0.04 | <0.001 |  | -0.02 | -0.04, -0.01 | 0.005 |  |
| Albumin, g/dL | 0.37 | 0.18, 0.56 | <0.001 |  | 0.10 | -0.09, 0.29 | 0.293 |  |
| LDL-C, mg/dL | 0.00 | 0.00, 0.00 | 0.713 |  | NS | . | . |  |
| Sodium, mmol/L | 0.01 | -0.01, 0.03 | 0.235 |  | NS | . | . |  |
| HbA1c, % | -0.01 | -0.08, 0.06 | 0.812 |  | NS | . | . |  |
| Hemoglobin, g/dL | 0.05 | 0.01, 0.09 | 0.008 |  | 0.00 | -0.04, 0.04 | 0.906 |  |
| hsCRP, mg/dL | -0.03 | -0.05, -0.01 | <0.001 |  | -0.01 | -0.02, 0.01 | 0.412 |  |
| ASM index, kg/m^2^ | 0.02 | -0.03, 0.07 | 0.399 |  | NS | . | . |  |
| Proteinuria, *n* (%) | -0.27 | -0.51, -0.03 | 0.028 |  | -0.16 | -0.41, 0.08 | 0.193 |  |

ASM, appendicular skeletal muscle mass; BMI, body mass index; eGFR, estimated glomerular filtration rate; GDS, Geriatric Depression Scale-Korean version; HbA1c, glycated hemoglobin; hsCRP, high-sensitivity C-reactive protein; LDL-C, low-density lipoprotein cholesterol.

**Supplementary Table 5 Simple and multiple linear regression analyses of word list recall scores in men**

|  | Simple | | |  | Multiple | | | |
| --- | --- | --- | --- | --- | --- | --- | --- | --- |
|  | estimate | 95% CI | *P* |  | estimate | 95% CI | *P* |  |
| log eGFR | 1.03 | 0.55, 1.51 | <0.001 |  | 0.51 | 0.00, 1.02 | 0.051 |  |
| Age, years | -0.07 | -0.08, -0.06 | <0.001 |  | -0.06 | -0.07, -0.04 | <0.001 |  |
| Men, *n* (%) | NS | . | . |  | NS | . | . |  |
| Education, years | 0.04 | 0.03, 0.05 | <0.001 |  | 0.03 | 0.02, 0.04 | <0.001 |  |
| Smoking, *n* (%) | -0.09 | -0.25, 0.08 | 0.304 |  | NS | . | . |  |
| Alcohol consumption, *n* (%) | 0.23 | 0.12, 0.34 | <0.001 |  | 0.15 | 0.04, 0.26 | 0.006 |  |
| BMI, kg/m^2^ | 0.05 | 0.03, 0.07 | <0.001 |  | 0.03 | 0.02, 0.05 | <0.001 |  |
| GDS score | -0.05 | -0.07, -0.03 | <0.001 |  | -0.02 | -0.04, -0.01 | 0.009 |  |
| Albumin, g/dL | 0.36 | 0.17, 0.55 | <0.001 |  | 0.10 | -0.09, 0.30 | 0.292 |  |
| LDL-C, mg/dL | 0.00 | 0.00, 0.00 | 0.029 |  | 0.00 | 0.00, 0.00 | 0.129 |  |
| Sodium, mmol/L | 0.01 | -0.01, 0.03 | 0.285 |  | NS | . | . |  |
| HbA1c, % | -0.03 | -0.10, 0.03 | 0.340 |  | NS | . | . |  |
| Hemoglobin, g/dL | 0.07 | 0.03, 0.10 | <0.001 |  | 0.00 | -0.04, 0.04 | 0.925 |  |
| hsCRP, mg/dL | -0.03 | -0.05, -0.02 | <0.001 |  | -0.01 | -0.03, 0.01 | 0.176 |  |
| ASM index, kg/m^2^ | 0.04 | -0.01, 0.09 | 0.154 |  | NS | . | . |  |
| Proteinuria, *n* (%) | -0.34 | -0.59, -0.09 | 0.007 |  | -0.18 | -0.43, 0.07 | 0.160 |  |

ASM, appendicular skeletal muscle mass; BMI, body mass index; eGFR, estimated glomerular filtration rate; GDS, Geriatric Depression Scale-Korean version; HbA1c, glycated hemoglobin; hsCRP, high-sensitivity C-reactive protein; LDL-C, low-density lipoprotein cholesterol.

**Supplementary Table 6 Simple and multiple linear regression analysis of MMSE**

|  | **Simple** | | |  | **Multiple** | | | |
| --- | --- | --- | --- | --- | --- | --- | --- | --- |
|  | estimate | 95% CI | *P* |  | estimate | 95% CI | *P* |  |
| log eGFR | 0.61 | 0.25, 0.98 | 0.001 |  | 0.23 | -0.10, 0.57 | 0.174 |  |
| Age, years | -0.06 | -0.07, -0.05 | <0.001 |  | -0.04 | -0.05, -0.03 | <0.001 |  |
| Men, *n* (%) | 0.37 | 0.30, 0.44 | <0.001 |  | -0.14 | -0.24, -0.04 | 0.007 |  |
| Education, years | 0.10 | 0.09, 0.11 | <0.001 |  | 0.09 | 0.09, 0.10 | <0.001 |  |
| Smoking, *n* (%) | 0.03 | -0.13, 0.19 | 0.711 |  | NS | . | . |  |
| Alcohol consumption, *n* (%) | 0.24 | 0.17, 0.31 | <0.001 |  | 0.05 | -0.01, 0.12 | 0.096 |  |
| BMI, kg/m^2^ | 0.01 | 0.00, 0.03 | 0.030 |  | 0.00 | -0.01, 0.01 | 0.960 |  |
| GDSK score | -0.07 | -0.08, -0.06 | <0.001 |  | -0.02 | -0.03, -0.01 | <0.001 |  |
| Albumin, g/dL | 0.35 | 0.21, 0.49 | <0.001 |  | 0.07 | -0.05, 0.20 | 0.253 |  |
| LDL-C, mg/dL | 0.00 | 0.00, 0.00 | 0.386 |  | NS | . | . |  |
| Sodium, mmol/L | 0.01 | -0.01, 0.02 | 0.319 |  | NS | . | . |  |
| HbA1c, % | 0.01 | -0.04, 0.06 | 0.697 |  | NS | . | . |  |
| Hemoglobin, g/dL | 0.13 | 0.10, 0.15 | <0.001 |  | 0.03 | 0.01, 0.06 | 0.014 |  |
| hsCRP, mg/dL | -0.02 | -0.04, -0.01 | 0.001 |  | -0.01 | -0.02, 0.01 | 0.345 |  |
| ASM index, kg/m^2^ | 0.13 | 0.09, 0.16 | <0.001 |  | 0.08 | 0.04, 0.12 | <0.001 |  |
| Proteinuria, *n* (%) | -0.01 | -0.22, 0.21 | 0.946 |  | NS | . | . |  |

ASM, appendicular skeletal muscle mass; BMI, body mass index; eGFR, estimated glomerular filtration rate; GDS, Geriatric Depression Scale-Korean version; HbA1c, glycated hemoglobin; hsCRP, high-sensitivity C-reactive protein; LDL-C, low-density lipoprotein cholesterol.

**Supplementary Table 7 Logistic regression analysis of the association between CKD stages and global cognitive impairment**

| Variables | No. of patients  /Total patients | Unadjusted | | |  | | Adjusted* | |  |
| --- | --- | --- | --- | --- | --- | --- | --- | --- | --- |
|  |  | OR (95% CI) | *P* |  | | OR (95% CI) | | *P* | |
| CKD stage 1 | 50/449 | 1.00 |  |  | | 1.00 | |  | |
| CKD stage 2 | 287/2038 | 1.312 (0.954, 1.806) | 0.095 |  | | 1.379 (0.988, 1.923) | | 0.059 | |
| CKD stage 3 | 51/335 | 1.433 (0.943, 1.806) | 0.092 |  | | 1.436 (0.925, 2.228) | | 0.107 | |
| CKD stage 4 | 8/22 | 4.560 (1.823, 11.409) | 0.001 |  | | 3.825 (1.387, 10.548) | | 0.010 | |
| CKD stage 5 | 0/3 | - | - |  | | - | | - | |

*Adjusted for smoking, alcohol consumption, body mass index, Geriatric Depression Scale-Korean version score, albumin, low-density lipoprotein, hemoglobin, proteinuria, and appendicular skeletal muscle mass index
